# Beyond the Visible and Below the Peel: How UV-B Radiation Influences the Phenolic Profile in the Pulp of Peach Fruit. A Biochemical and Molecular Study

**DOI:** 10.3389/fpls.2020.579063

**Published:** 2020-10-30

**Authors:** Marco Santin, Antonella Castagna, Begoña Miras-Moreno, Gabriele Rocchetti, Luigi Lucini, Marie-Theres Hauser, Annamaria Ranieri

**Affiliations:** ^1^Department of Agriculture, Food and Environment, University of Pisa, Pisa, Italy; ^2^Interdepartmental Research Center Nutrafood “Nutraceuticals and Food for Health”, University of Pisa, Pisa, Italy; ^3^Department for Sustainable Food Process, Università Cattolica del Sacro Cuore, Piacenza, Italy; ^4^Council for Agricultural Research and Economics- Research Centre for Genomics and Bioinformatics, Fiorenzuola d’Arda, Italy; ^5^Department of Applied Genetics and Cell Biology, University of Natural Resources and Life Sciences, Vienna, Austria

**Keywords:** UV-B radiation, peach fruit, *Prunus persica*, phenolics, flavonols, anthocyanins

## Abstract

In the last decades, UV-B radiation has attracted attention due to its potential to increase nutraceutical values of fruit and vegetables, especially by inducing the accumulation of phenolics in a structure-dependent way. However, most current studies have investigated the UV-B-driven changes only in the peel or focusing on individual phenolic classes. Adopting an “-omics” approach, this work aimed to deepen the knowledge about the effects of UV-B radiation on the phenolic profile in the pulp of peach fruit. Based on these considerations, melting flesh yellow peaches (*Prunus persica* L., cv. Fairtime) were subjected to either a 10- or 60-min UV-B treatment (1.39 and 8.33 kJ m^–2^, respectively), and sampled at different time points from the exposure. A UHPLC-ESI/QTOF-MS analysis coupled with a phenolics-specific database for the annotation of compounds and a multivariate discriminant analysis revealed a marked effect of UV-B radiation on the phenolic profiles of peach pulp. Particularly, a general, transient increase was observed after 24 h from the irradiation, especially for flavanols, flavonols, and flavones. Such behavior diverges from what was observed in the peel, where an overall increase of phenolics was observed after 36 h from the irradiation. Concerning the flavonols in the pulp, UV-B exposure stimulated a specific accumulation of isorhamnetin and kaempferol derivatives, with variations imposed by the different sugar moiety bound. Anthocyanins, which were the second most abundant flavonoid group after flavonols, displayed a general decrease after 36 h that was not attributable to specific molecules. The UV-B treatments also increased the glycoside/aglycone ratio of flavonols and anthocyanins after 24 h, by increasing the glycoside concentration of both, flavonols and anthocyanins, and decreasing the aglycone concentration of anthocyanins. In support of the biochemical results, targeted gene expression analysis by RT-qPCR revealed an UV-B-induced activation of many genes involved in the flavonoid pathway, e.g., CHS, F3H, F3′H, DFR, as well as some MYB transcription factors and few genes involved in the UV-B perception. Generally, all the flavonoid-related and MYB genes showed a transient UV-B dose-dependent activation after 6 h from the irradiation, similarly to what was observed in the peel.

## Introduction

Peach (*Prunus persica* L.), originally domesticated in China, has become a highly appreciated fruit all over the Globe, with particular spread throughout the Mediterranean countries due to the favorable environmental conditions for its cultivation (Konopacka et al., 2010). The great popularity of peach fruit among consumers derives from both its appreciated organoleptic traits and its high nutraceutical value, thanks to the elevated content of health-promoting compounds such as polyphenols, carotenoids, and ascorbic acid (Tomás-Barberán et al., 2001; Gil et al., 2002).

Phenolic compounds represent a huge class of bioactive secondary plant metabolites, which fulfill essential functions during the lifespan of plants such as growth, reproduction, acclimation and defense (Ghasemzadeh and Ghasemzadeh, 2011; Zhang and Tsao, 2016). According to their chemical structure, functions and biosynthesis, phenolic compounds can be classified in flavonoids, lignans, phenolic acids, stilbenes, and other lower-molecular-weight compounds.

Flavonoids comprehend more than 6000 members, and surely play a key role in many defense mechanisms toward both biotic and abiotic stresses, since they possess the highest antioxidant and metal chelating activities in the phenolic group (Tolonen et al., 2002; Austin and Noel, 2003; Cheynier et al., 2013). They are commonly categorized into flavonols, isoflavonoids, flavones, flavanones, flavanols, and anthocyanidins (Ross and Kasum, 2002; Herndon et al., 2018). In plants, they are mainly glycosylated through the phenolic hydroxyls with one or multiple sugar moieties (Xiao et al., 2014; Tohge et al., 2017). The main flavonoids detected in the peach fruit are flavonols, flavan-3-ols and anthocyanins, whose concentration is strictly related to environmental factors and farming practices (Tomás-Barberán et al., 2001; Vizzotto et al., 2007; Tavarini et al., 2011; Aleixandre et al., 2013; Mokrani et al., 2016). Flavonoids provide protection against solar high-energy radiations, such as ultraviolet (UV), which can potentially damage macromolecules in plant cells through the overproduction of reactive oxygen species (ROS) (Frohnmeyer, 2003; Zhang et al., 2006; Hideg et al., 2013; Jenkins, 2014). UV-B comprehends a narrow wavelength range (280–315 nm) of the total UV radiation, whose majority is blocked by the stratospheric ozone shielding (Nunez et al., 1994; Herndon et al., 2018).

To achieve this acclimation response, UV-B activates a specific intracellular pathway mediated by the UV RESISTANCE LOCUS 8 (UVR8), a dimeric, inactive, cytoplasmic protein which acts as a UV-B-photoreceptor (Rizzini et al., 2011). Once UV-B hits UVR8, it rapidly monomerizes and the monomer interacts with the E3 ubiquitin ligase CONSTITUTIVELY PHOTOMORPHOGENIC 1 (COP1) (Favory et al., 2009). Interaction of activated UVR8 with COP1 leads to accumulation of COP1 target proteins, such as the bZIP transcription factor ELONGATED HYPOCOTYL 5 (HY5) (Favory et al., 2009; Huang et al., 2013; Lau et al., 2019). Stabilized HY5 is crucial for the regulation of numerous UV-B-induced genes, including those important for acclimation to UV-B (Ulm et al., 2004; Brown et al., 2005; Brown and Jenkins, 2008; Gruber et al., 2010; Stracke et al., 2010; Binkert et al., 2014). The expression of HY5 is also enhanced by the action of UVR8-COP1 complex, which in turn acts by over-expressing many genes involved in UV-B acclimation (Brown et al., 2005; Favory et al., 2009). Among the responsive flavonoid biosynthetic genes, *CHALCONE SYNTASE* (*CHS*), *CHALCONE ISOMERASE* (*CHI*), *FLAVANONE 3-HYDROXYLASE* (*F3H*), *DIHYDROFLAVONOL 4-REDUCTASE* (*DFR*), *ANTHOCYA NIDIN SYNTHASE* (*ANS*), and *UDP-GLUCOSE:FLAVONOID 3-O-GLUCOSYLTRANSFERASE* (*UFGluT*) were found to be upregulated, together with an increase of phenolic compounds, in many fruit species such as apple (Ubi et al., 2006), tomato (Catola et al., 2017), and peach (Scattino et al., 2014; Santin et al., 2019). However, the previously cited works studied only the peel, i.e., the tissue directly exposed to UV-B radiation and thus more likely to be influenced by the treatment.

To the best of our knowledge, no studies have explored the UV-B-driven molecular and biochemical changes on the pulp of UV-B-irradiated fruit. The present work aimed to deeply investigate whether UV-B radiation influences the flavonoid profile of peach pulp and if these changes were accompanied by UVR8-dependent expression of flavonoid biosynthetic and regulatory genes.

## Materials and Methods

### Plant Material and UV-B Irradiation

A set of organic peach fruit (*Prunus persica* L., cv. Fairtime) was bought in an organic supermarket and meticulously chosen to be undamaged and similar in size, color, and dimension (8.1 cm average diameter). Groups of five peaches were randomly assigned to either controls or UV-B-treated (10- or 60-min UV-B treatment) sets. UV-B exposure was conducted at 24°C in climatic chambers, each equipped with four UV-B tubes (Philips Ultraviolet-B Narrowband, TL 20W/01– RS, Koninklijke Philips Electronics, Eindhoven, Netherlands) and white light tubes (Philips F17T8/TL741). The 10- and 60-min UV-B treated fruit were subjected to a total irradiance of 6.42 kJ m^–2^ (1.39 kJ m^–2^ UV-B + 5.03 kJ m^–2^ white light) and 38.53 kJ m^–2^ (8.33 kJ m^–2^ UV-B + 30.20 kJ m^–2^ white light), respectively, at fruit height. After the UV-B irradiation, controls and UV-B-exposed peaches were kept under white light at room temperature and sampled after 6, 12, 24, and 36 h from the end of the treatment for the molecular analysis, and after 24 and 36 h for the biochemical analysis. A portion of peach pulp (1.5 cm thick just below the skin, corresponding to approximatively one third of the fruit thickness from peel to pit) from the UV-B-exposed area was sampled with scalpels and tweezers, immediately frozen in liquid nitrogen and stored at −80°C until further analyses.

### RNA Extraction and cDNA Synthesis

RNA extraction was conducted from freeze-dried peach pulp using the LiCl/CTAB protocol (Richter et al., 2017) slightly modified. Briefly, 50 mg of lyophilized pulp were finely ground and 3 mL of pre-heated RNA extraction buffer (2% [w/v] hexadecyltrimethylammonium bromide, CTAB; 2% [w/v] polyvinylpyrrolidone, PVP; 100 mM Tris/HCl pH 8.0; 25 mM EDTA; 2M NaCl; 0.5 g/L spermidine and 2.7% [v/v] 2-mercaptoethanol) were added. After an incubation of 5 min at 65°C, 3 mL of ice-cold chloroform:isoamylalcohol (24:1) were added and mixed well for 5 min. The suspension was centrifuged at 4250 *g* for 20 min at 4°C and the supernatant was washed with ice-cold chloroform:isoamylalcohol (24:1) followed by another centrifugation step. RNA precipitation was achieved by adding ice-cold 10M LiCl, and the samples were kept at 4°C overnight. By centrifuging (12000 *g* for 1 h at 4°C), the RNA pellet was isolated and then washed with 75% EtOH, dissolved in 30 μL RNAse free water and stored at −80°C. Qubit (Invitrogen) and the NanoDrop systems were used for RNA quantification, and the integrity of the isolated RNA was checked by separation on a 1.2% agarose gel. To avoid genomic DNA contamination, the RNA was incubated with 1 U RNase-free DNaseI (Fermentas) in the presence of 25 mM MgCl2 at 37°C for 30 min, as stated by Karsai et al. (2002). Reverse transcription was carried out using 1 μL of peqGOLD M-MuLV H Plus, 200 U/μL (Peqlab), in a RT master mix containing 5 x RT buffer (provided with RT enzyme), 1 mM dNTP and 50 pMol oligo(dT)18. Samples were incubated for 60 min at 37°C, then the reaction was stopped at 75°C for 5 min. The obtained cDNA was diluted five times with sterile double-distilled water, and stored at −20°C.

### Real Time Quantitative PCR (RT-qPCR)

Three reference gene candidates were primarily tested [*EUKARYOTIC INITIATION FACTOR-4A* (*PpEIF4A*), *TUBULIN BETA-9 CHAIN* (*PpTUB9*), and *UBIQUITIN 5* (*PpUBQ5*)]. The threshold cycles (Ct values) for each reference gene candidate in our groups of samples are reported in Supplementary Table S1. NormFinder software was used to calculate the expression stability value (M) in our sample set and for selecting the most suitable reference gene (Andersen et al., 2004). According to NormFinder, *PpEIF4A* was the most stable expressed reference gene candidate (M for *PpEIF4A*: 0.032; for *PpTUB9*: 0.159; for *PpUBQ5*: 0.202). Therefore, *PpEIF4A* was used to normalize all the RT-qPCR data. The latest GenBank database and the Genome Database for Peach *Prunus persica* genome^1^ were consulted to find suitable primers for *PpEIF4A*, *PpCHS*, *PpF3H*, *PpF3′H*, *PpDFR*, *PpMYB111*, *PpMYB-like*, *PpCOP1*, *PpHY5*, *PpUVR8*, based on BLAST alignments to the corresponding Arabidopsis genes as starting point (Altschul et al., 1997). The full list of primer sequences refers to Santin et al. (2019) and is reported as Supplementary Table S2. The Rotorgene-3000 cycler (Corbett, Qiagen, Germany) was used for the RT-qPCR runs. Each RT-qPCR reaction, performed in triplicate, was assembled in a total volume of 14 μL using the 5x HOT FIREPol EvaGreen^®^ qPCR Mix Plus (Solis BioDyne, Tartu, Estonia). 2.8 μL of 5x HOT FIREPol EvaGreen^®^ qPCR Mix were mixed with 0.25 μL of forward and reverse primers (each 20 μM), 1 μL of the cDNA template, and double-distilled water. The cDNA was initially denatured at 95°C for 12 min and then amplified [40 cycles: 55°C/5 s, 67°C/25 s (extension and acquisition in channel A), 76°C/6 s (acquisition in channel B), 82°C/6 s (acquisition in channel C), and denaturation at 95°C/5 s]. In addition, a standard curve using serial diluted templates (from 10^7^ to 10^2^, with 10^5^, 10^4^, and 10^3^ in duplicate, and a blank) was created for each gene, to determine the PCR efficiency (Supplementary Table S2). In each PCR reaction, a standard curve was also inserted. Integrated Rotorgene software was used to calculate the number of copies/μL based on the gene specific standard curves, and normalization was performed with respect to the *PpEIF4A* reference gene copy number. For each gene, standard curves of known PCR amplicon copy number were designed, and serial dilutions of quantified PCR fragment were included in each run and the PCR efficiencies determined. RT-qPCR data represent means and standard errors of five independent biological replicates.

### Phenolic Extraction and UHPLC-ESI-QTOF-MS Analysis

Phenolic compounds were extracted from peach pulp by using a homogenizer-assisted extraction (Santin et al., 2019). In particular, samples (1.0 g) were homogenized in 10 mL of hydro-alcoholic solution (i.e., methanol 80% v/v, acidified with 0.1% HCOOH) by using an Ultra-turrax (Ika T25, Staufen, Germany), at maximum speed for 3 min. Following centrifugation (6,000 × *g*, for 10 min at 4°C), the extracts were filtered in amber vials using 0.22 μm cellulose syringe filters, to be analyzed by ultra-high-pressure liquid chromatography (UHPLC) coupled with quadrupole time-of-flight (QTOF) mass spectrometry. In this regard, the instrumental conditions for the analysis of phenolic compounds were previously optimized (Rocchetti et al., 2019). Briefly, chromatographic separation was done in reverse phase mode, using an Agilent Zorbax Eclipse Plus C18 column (100 mm, 1.9 μm particle size). A binary mixture of acetonitrile and water (both acidified with 0.1% HCOOH) was used as mobile phase, with a gradient from 6% acetonitrile up to 94% acetonitrile in 32 min. Also, the mobile phase temperature was set to 35°C, with a flow rate of 220 μL min^–1^ and an injection volume of 6 μL. Regarding the high-resolution mass spectrometry analysis, the QTOF worked in positive (ESI +) MS-only mode, acquiring accurate masses in the 100–1200*m/z* range at a rate of 0.8 spectra/s. The QTOF was operated in dynamic range mode with a nominal mass resolution of 30,000 FWHM. Nitrogen was used as both sheath gas (10 L min^–1^ at 350°C) and drying gas (8 L min^–1^ at 330°C). The nebulizer pressure was 60 psig, nozzle voltage was 300 V, and capillary voltage was 3.5 kV. The identification of polyphenols was achieved using the software Profinder B.07 from Agilent Technologies, according to the “find-by-formula” algorithm. In particular, the annotations were recursively done against the comprehensive database Phenol-Explorer^2^ and using the entire isotopic profile (including isotopic spacing and isotopic ratio, with a maximum of 5 ppm for monoisotopic mass accuracy). Therefore, a Level 2 of compound identification was achieved as set out by the COSMOS Metabolomics Standards Initiative (Salek et al., 2013; Schrimpe-Rutledge et al., 2016). The obtained dataset was further used for statistics and chemometrics. Finally, semi-quantitative information regarding the main flavonoids’ subclasses were obtained by using representative standard compounds (Rocchetti et al., 2018): cyanidin (anthocyanins), catechin (flavan-3-ols and flavonols), and luteolin (flavones and other remaining subclasses), were used with this aim. Three replicates were processed (*n* = 3), and results were finally expressed as mg kg^–1^ dry weight (DW).

### Statistical Analysis

Firstly, a canonical discriminant analysis (CDA) was performed starting from the whole phenolics dataset, to check the effectiveness of the UV-B treatments in modulating the phenolic concentration in both recovery time points tested. Besides, all biochemical and molecular data were analyzed by one-way ANOVA followed by Tukey–Kramer *post hoc* test at the significance level *P* ≤ 0.05 to evaluate which phenolic subclasses are mainly responsive to the UV-B treatments. To elaborate and discuss the results, the recovery timepoints were deliberately kept separated to avoid the possible hiding effect of the physiological phenolic modification due to the fruit ripening process on the phenolic changes due to the UV-B exposure. All the statistical analyses were performed using JMP software (SAS Institute, Inc., Cary, NC, United States). Regarding the statistical elaboration of metabolomics-based data, the software Agilent Mass Profiler Professional B.12.06 (from Agilent Technologies) was used. In particular, phenolic compounds were filtered by abundance (min. abundance 10,000, roughly corresponding to a signal-to-noise of 5) and by frequency (retaining the compounds present in 100% of samples in at least one condition), normalized at the 75^*th*^ percentile and baselined to the median of each compound in the dataset.

## Results

### UV-B-Irradiation Rearranged the Phenolic Profile in Peach Pulp

**FIGURE 1:**
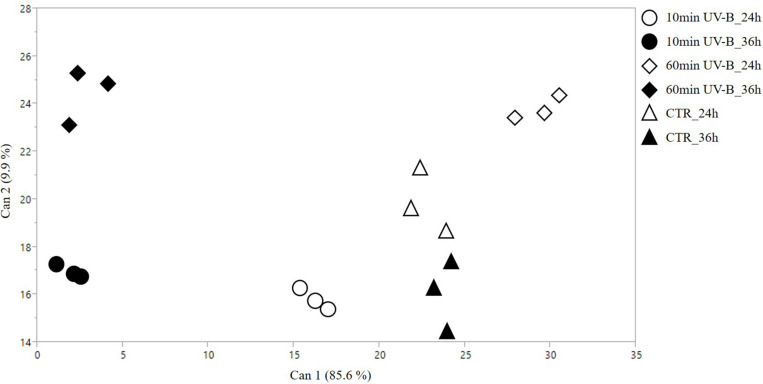
2D scatterplot of canonical discriminant analysis (CDA) considering the samples treated with 10 and 60 min of UV-B radiation after 24 and 36 h from the exposure, as well as the UV-B-untreated samples [controls (CTR)]. The whole phenolics dataset was used to perform this analysis. Can 1 and 2 refers to the canonical functions 1 and 2, which include all the variables to maximize the separation among the groups.

The untargeted metabolomic approach based on UHPLC-QTOF mass spectrometry allowed us to putatively annotate more than 420 phenolic compounds, which were then classified in their respective phenolic subclass. This was done to better describe the UV-B-induced effects as related to their structure and function. A comprehensive list containing all phenolics identified together with their composite mass spectrum is reported in Supplementary Table S3. Overall, flavonoids were found to be the most represented phenolic class, accounting for 52% of the total phenolics, followed by phenolic acids (86 compounds, 79 tyrosol equivalents, 29 lignans, and 10 stilbenes). The first step of this work was to check whether the UV-B exposures were able to induce an overall modification of the phenolic profile of both recovery time points. To this aim, a multivariate technique, the CDA, was performed including both the UV-B treatments and recovery time points (Figure 1). CDA represents a dimension-reduction statistical tool, which is strictly associated with the principal component analysis (PCA) but, unlike the PCA, it is usually performed when the different groups are known from the very beginning. In particular, the model tends to maximize the differences among the preassigned groups, maximizing at the same time the similarities in each group.

The CDA reported in Figure 1 clearly showed that biological replicates fit the preassigned groups according to the phenolic dataset, with no outliers detected in the hyperspace. Robustness of this statistical analysis was assessed with four MANOVA tests (Wilk’s Lambda, Roy’s Largest Root Test, the Hotelling-Lawley Trace and the Pillai-Bartlett Trace), which all gave highly significant *P*-values (i.e., <0.0001). The canonical functions 1 and 2 explained 85.6 and 9.9% of the segregation, respectively. The projections of the two control groups on the canonical function 1 were overlapping, indicating that no differences are appreciable on the first canonical variable. Similarly, the 10- and the 60-min UV-B treated groups after 36 h are located on the far left of the scatterplot. The UV-B-exposed groups after 24 h, however, were the only ones that displayed a marked segregation, being the 10- and the 60-min UV-B treated groups located at the opposite sides of the control groups. Concerning the canonical function 2, the 60-min UV-B-treated groups are distributed in the upper part of the plot, being well-separated from the others. Below, the controls after 24 h do not coincide with any other set of samples, while the 10-min UV-B-treated groups, both after 24 and 36 h, and the controls after 24 h, are placed at the lowest part of the plot. The great separation of the groups in the hyperspace, especially between the UV-B-treated ones and the control ones considering the canonical 1, suggested a strong UV-B-induced modification of the phenolic profile in the pulp of peach fruit, which deserved a deepen investigation.

### UV-B-Induced Flavonoid Modifications Involved Different Subclasses at Distinct Recovery Time Points

**FIGURE 2:**
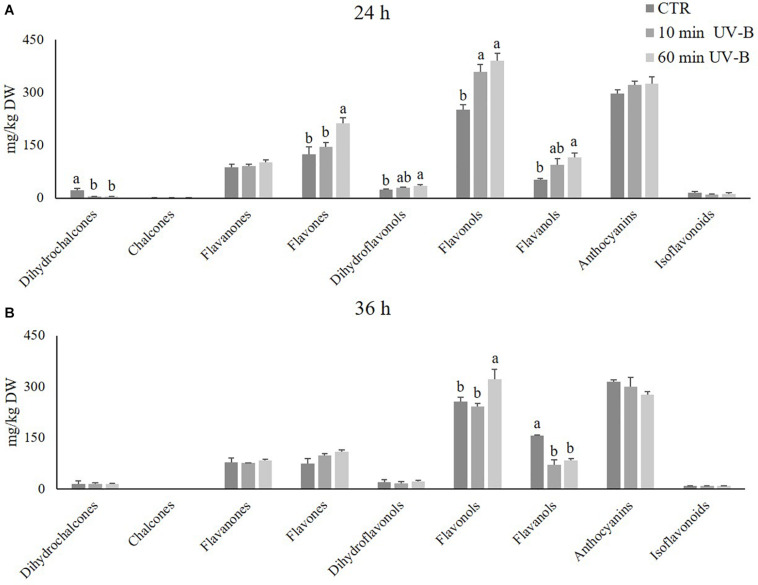
Concentration (mg/kg DW) of several flavonoid classes (dihydrochalcones, chalcones, flavanones, flavones, dihydroflavonols, flavonols, flavanols, anthocyanins, and isoflavonoids) in the pulp of peaches exposed to UV-B radiation for either 10 or 60 min, and sampled after 24 h **(A)** and 36 h **(B)** from the exposure. Data are mean ± SE of five biological replicates. Different letters correspond to statistically significant differences according to one-way ANOVA followed by Tukey–Kramer *post hoc* test (*P* ≤ 0.05).

Our next step was to investigate further which phenolic subclasses were mainly responsive to the UV-B treatments, and whether the UV-B exposure might induce positive or negative modifications on such phenolic subclasses. Particularly, the attention was focused on the flavonoid family that, possess the highest antioxidant activity among phenolics and were more likely to be influenced by the UV-B radiation. The whole phenolic dataset was then rearranged combining all the compounds in their respective phenolic subclasses, and the total concentration for each subclass was calculated by summing the individual concentrations of its members. Results are graphically presented in Figure 2. At a first glance, it was evident that flavonols and anthocyanins represented the most abundant flavonoid subclasses in the pulp of peaches, with average concentrations in control samples of 251 and 298 mg/kg and 257 and 316 mg/kg after 24 and 36 h of incubation under white light only, respectively. Afterward, flavanols, flavones, and flavanones were the most highly concentrated ones, with average concentrations of 104, 100, and 83 mg/kg, respectively.

The UV-B treatments triggered modifications in the flavonoid profile according to the recovery times, indicating that the UV-B-induced effects were still detectable after several hours of storage. Particularly after 24 h from both the 10- and 60-min UV-B exposures (Figure 2A), the most responsive flavonoid classes were flavanols, flavones, flavonols, and dihydroflavonols. All these classes showed a significant increase when peaches were exposed to 60 min of UV-B (+ 123, + 70, + 55, and + 50% compared to the control group, respectively), suggesting a positive role of the UV-B radiation in enhancing such flavonoid subfamilies. Besides, the 10-min UV-B exposure was also significantly effective when considering the flavonols, which were + 43% more concentrated in the UV-B-treated group. An increasing trend occurred also regarding flavanols, flavones, and dihydroflavonols irradiated for 10 min, suggesting a dose-dependent response. The only flavonoids undergoing a decrease in both UV-B-treated groups were the dihydrochalcones.

After 36-h of recovery (Figure 2B), the responsive flavonoid subclasses were flavonols and flavanols. Regarding flavonols, the 60-min treatment was still effective in maintaining their concentration significantly higher than the controls (+ 52%). Flavanols, however, responded contrarily to what was observed after 24 h from the UV-B exposure. Indeed, both the UV-B-treated groups exhibited a significantly lower flavanol concentration than the controls (-55 and -47% for the 10- and the 60-min treated groups, respectively).

### UV-B Radiation Was Effective in Increasing Flavonol Concentration in a Structure-Dependent Way Only After 24 h From UV-B Exposure

**FIGURE 3:**
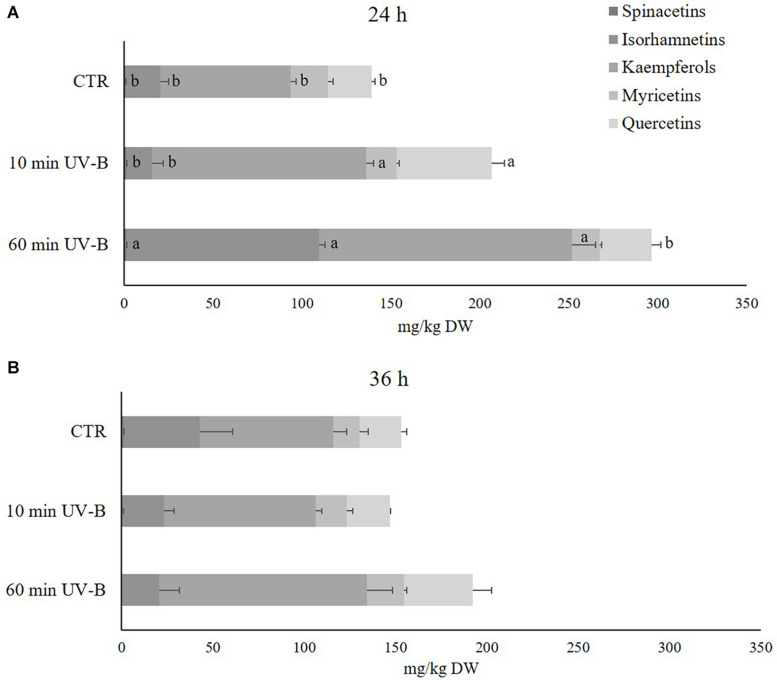
Concentration (mg/kg DW) of different flavonols (spinacetins, isorhamnetins, kaempferols, myricetins, and quercetins) in the pulp of peaches exposed to UV-B radiation for either 10 or 60 min and sampled after 24 h **(A)** and 36 h **(B)** from the exposure. Data are mean ± SE of five biological replicates. Different letters correspond to statistically significant differences according to one-way ANOVA followed by Tukey–Kramer *post hoc* test (*P* ≤ 0.05).

Since flavonols were the most abundant flavonoid class in peach pulp, thus contributing the most to the fruit quality, the next step was to investigate deeper the UV-B response of the different flavonols at the 24 and 36 h recovery time points (Figure 3). Sixty-one different flavonols were identified (Supplementary Table S3), including glycosides and aglycones, and they were grouped in relation to the respective flavonol moiety. Accordingly, 3 isorhamnetins, 18 kaempferols, 5 myricetins, 21 quercetins, and 2 spinacetins were found. Flavonols with less than two derivatives were not considered in this analysis since they were not likely contributing to the overall flavonol response. Kaempferols resulted to be the most abundant flavonol subclass in the peach pulp, with an average concentration in control samples of 72.9 mg/kg in both recovery time points, followed by isorhamnetins and quercetins (19.8 and 24.6, 41.8 and 22.8 mg/kg after 24 and 36 h, respectively). As observed in Figure 3A, after 24 h of recovery, the 60-min UV-B exposure was effective in increasing the concentration of spinacetins, isorhamnetins, kaempferols (+ 61, + 448, and + 95%, respectively). A not significant tendency of increased quercetin was also detected. The 10-min UV-B treatment did not induce such accumulation of flavonols, instead being successful to determine a significant increase only for quercetins and kaempferols (+ 118 and + 66%, respectively). However, after the 36-h recovery time point (Figure 3B), no significant variation was observed in both the UV-B treatments for any of the flavonol subclasses detected. Since a significant accumulation after 36 h from the 60-min UV-B exposure was found for the whole class of flavonols (Figure 2B), it meant that the individual flavonols subclasses underwent a slight, not significant, increase, whose sum however resulted to be significantly higher compared to the controls.

#### Kaempferol and Isorhamnetins Glycosides Responded Differently to UV-B Radiation in Relation to Their Sugar Moiety

**FIGURE 4:**
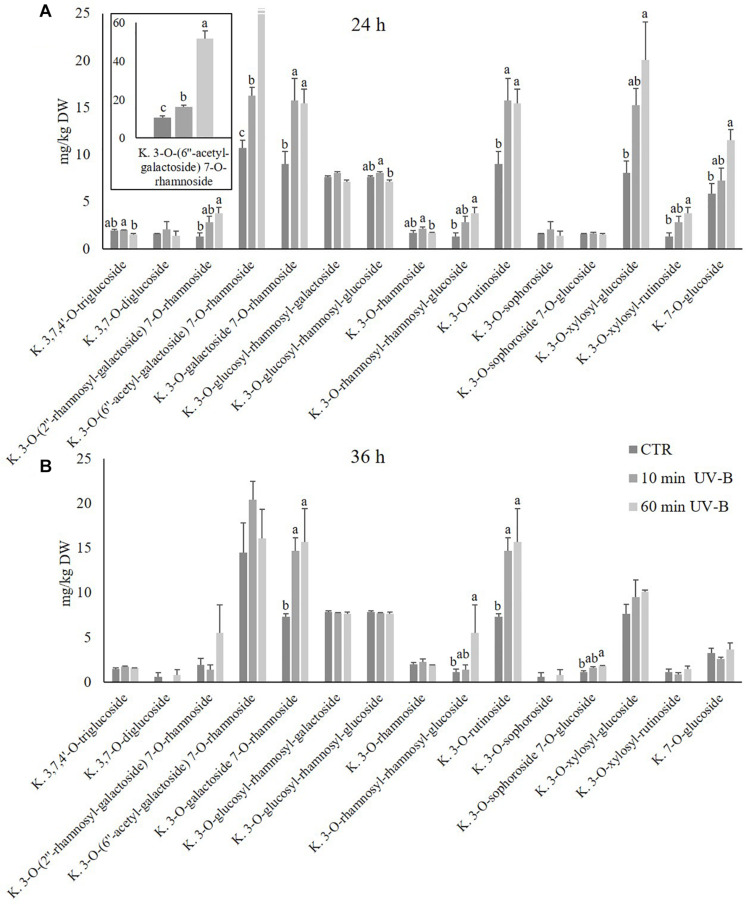
Concentration (mg/kg DW) of different kaempferols in the pulp of peaches exposed to UV-B radiation for either 10 or 60 min and sampled after 24 h **(A)** and 36 h **(B)** from the exposure. **(A)** Kaempferol 3-*O*-(6′′-acetyl-galactoside) 7-*O*-rhamnoside was reported also in a different, framed histogram with a higher y-axis scale (0–60 mg/kg DW) for a better visualization. Data are mean ± SE of five biological replicates. Different letters correspond to statistically significant differences according to one-way ANOVA followed by Tukey–Kramer *post hoc* test (*P* ≤ 0.05).

**FIGURE 5:**
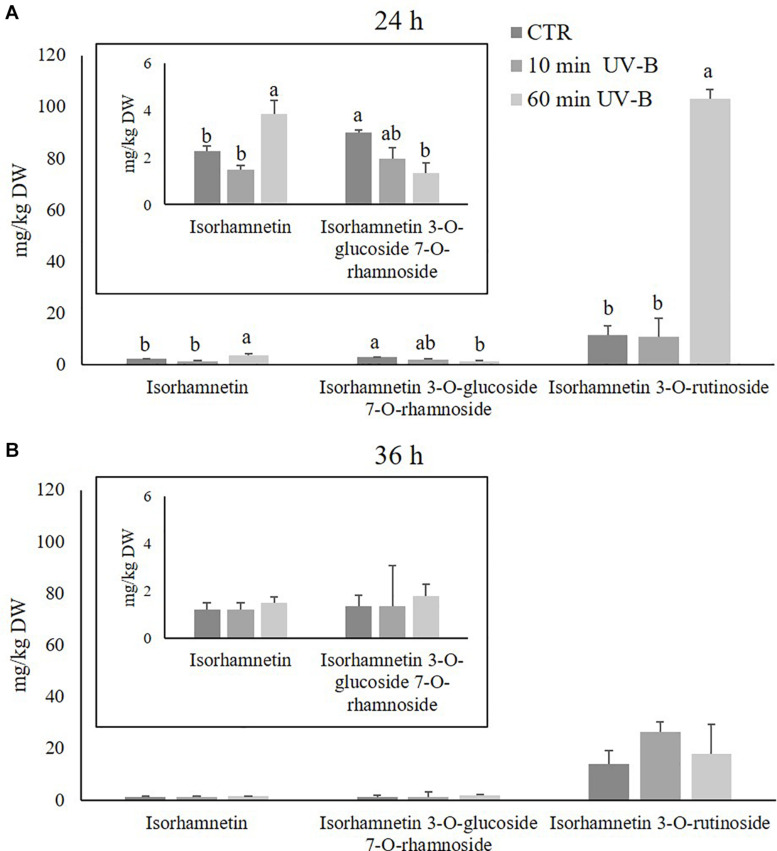
Concentration (mg/kg DW) of different isorhamnetins in the pulp of peaches exposed to UV-B radiation for either 10 or 60 min and sampled after 24 h **(A)** and 36 h **(B)** from the exposure. **(A)** Isorhamnetin and isorhamnetin 3-*O*-glucoside 7-*O*-rhamnoside were reported also in a different, framed histogram with a lower y-axis scale (0–6 mg/kg DW) for a better visualization. Data are mean ± SE of five biological replicates. Different letters correspond to statistically significant differences according to one-way ANOVA followed by Tukey–Kramer *post hoc* test (*P* ≤ 0.05).

Since kaempferols were found to be the most abundant flavonols detected, and since they were highly UV-B-responsive in the 24-h time point after both UV-B treatments, a further exploration was conducted to study the response of individual kaempferol glycosides. As observed in Figure 4, **15** different kaempferol glycosides were identified, underlying the complexity of the flavonols in the pulp. All the kaempferols detected were O-glycosidically linked to sugar moieties at the C3 and/or C7 position, and most of them were di- or tri-glycosides. The most abundant kaempferols of control samples were 3-*O*-(6′′-acetyl-galactoside) 7-*O*-rhamnoside, 3-*O*-galactoside 7-*O*-rhamnoside, 3-*O*-rutinoside and 3-*O*-xylosyl-glucoside, with concentrations in control samples of 10.7, 9.0, 9.0, and 8.1 mg/kg and 14.5, 7.4, 7.4, and 7.6 mg/kg after 24 and 36 h. When peaches were treated with UV-B, behavior of kaempferols differed considerably according to the substituents linked to the kaempferol moiety. After 24 h from the irradiation (Figure 4A), several kaempferols underwent an increase which was mostly UV-B-dose-dependent. In particular, the 60-min UV-B exposure was effective in increasing the concentration of the 3-*O*-(2′′-rhamnosyl-galactoside) 7-*O*-rhamnoside (+ 189%), the 3-*O*-(6′′-acetyl-galactoside) 7-*O*-rhamnoside (+ 381%), the 3-*O*-galactoside 7-*O*-rhamnoside (+ 71%), the 3-*O*-rhamnosyl-rhamnosyl-glucoside (+ 192%), the 3-*O*-rutinoside (+ 73%), the 3-*O*-xylosyl-glucoside (+ 149%), the 3-*O*-xylosyl-rutinoside (+ 190%), and the 7-*O*-glucoside (+ 97%). The 10-min UV-B exposure was less effective in inducing the accumulation of kaempferols, being successful for the 3-*O*-(6′′-acetyl-galactoside) 7-*O*-rhamnoside (+ 52%), the 3-*O*-galactoside 7-*O*-rhamnoside (+ 75%), and the 3-*O*-rutinoside (+ 52%). After 36 h from both the UV-B exposures (Figure 4B), only few significant changes were observed. In detail, the 60-min UV-B treatment induced a significant accumulation of the 3-*O*-galactoside 7-*O*-rhamnoside (+ 114%), the 3-*O*-rhamnosyl-rhamnosyl-glucoside (+ 371%), the 3-*O*-rutinoside (+ 115%), and the 3-*O*-sophoroside 7-*O*-glucoside (+ 61%). The 10-min UV-B irradiation, as observed in the 24-h recovery time point, stimulated a minor increase in terms of entity and number of kaempferols affected as compared to the 60-min UV-B irradiation. Indeed, only the 3-*O*-galactoside 7-*O*-rhamnoside and the 3-*O*-rutinoside were positively affected by the 10-min irradiation, with an increase of 100 and 102%, respectively. In addition, no kaempferols underwent a decrease following UV-B irradiation at any dose and any recovery time points, compared to the respective control. Like the kaempferols, the isorhamnetins were studied more in detail (Figure 5), since they underwent a massive increase after 24 h from the 60-min UV-B treatment (+ 448%). This flavonoid subclass comprised three molecules, the most abundant being a monoglycoside, the 3-*O*-rutinoside, followed by 3-*O*-glucoside 7-*O*-rhamnoside and the aglycone isorhamnetin. As observed for the kaempferols, the UV-B radiation induced more significant changes in the isorhamnetin concentration after 24 h then after 36 h of recovery. Considering the 24-h time point (Figure 5A), the 10-min UV-B exposure was ineffective in determining significant modifications of any of the isorhamnetin detected, while the 60-min irradiation induced an increase of the 3-*O*-rutinoside (+ 804%) and the aglycone (+ 70%). Since the 3-*O*-rutinoside was the most concentrated isorhamnetin and also the most UV-B-responsive one, it represented the main contributor to the overall increase in total isorhamnetins observed following UV-B exposure (Figure 3A). In the same recovery time point, the 60-min UV-B irradiation also determined a slight decrease in the 3-*O*-glucoside 7-*O*-rhamnoside (-56%). However, such decrease did not affect the total isorhamnetin due to the low concentration of this compound. Similar to the total flavonols and kaempferols, both UV-B exposures did not induce variations in any of the isorhamnetins at the 36-h recovery time point.

#### Total Flavonol Glycosides, but Not Aglycones, Transiently Responded to UV-B Radiation

Another interesting behavior relates the UV-B-responsiveness of the flavonol glycosides and aglycones. After having separated the aglycones (11) from the glycosides (54) in the dataset, the total concentrations for each group was calculated (Table 1). The concentration of glycosides was significantly higher than that of the aglycones, regardless of the UV-B treatment and the recovery time. Specifically, the glycoside concentration in the control groups was 21 and 45% higher than that of the aglycone in the 24 and 36 h recovery time points, respectively. As observed for the total and individual flavonols, the UV-B effects were more visible after 24 h from the irradiation also when considering the overall glycoside and aglycone concentrations. At this recovery time point, as reported in Table 1, both the 10- and the 60-min irradiations were effective in significantly inducing the accumulation of flavonol glycosides by 66 and 92%, respectively. On the other hand, no UV-B-induced variations in the aglycone concentrations were observed. Thus, the glycosides/aglycones ratio increased from 1.2 for the controls, up to 1.9 and 2.8 in the 10- and 60-min UV-B-irradiated fruit. No variations were observed for both, the glycosides and the aglycones after 36 h from the UV-B exposures.

**TABLE 1:** Concentration of total flavonol aglycones and glycosides in the UV-B-treated samples after 24 and 36 h from the UV-B exposure.

		Aglycones	Glycosides

		(mg/kg DW)	(mg/kg DW)
24 h	CTR	113.6 ± 8.3b	137.5 ± 10.6*Ba*
	10 min UV-B	121.3 ± 3.5b	228.3 ± 41.5*Aa*
	60 min UV-B	92.8 ± 10.8b	263.5 ± 25.2*Aa*
36 h	CTR	105.0 ± 18.2b	152.2 ± 9.0a
	10 min UV-B	97.1 ± 18.4b	145.6 ± 7.4a
	60 min UV-B	113.1 ± 15.4b	180.9 ± 49.9a

*Data are mean ± SE of three biological replicates. Different letters correspond to statistically significant differences according to one-way ANOVA followed by Tukey–Kramer *post hoc* test (*P* ≤ 0.05). Lowercase letters refer to the comparison between aglycones and glycosides, while uppercase letters refer to the comparison between the controls and the UV-B-treated groups in each recovery time point (24 or 36 h).*

### Total Anthocyanins Were Not Affected by UV-B Radiation While Individual Anthocyanins Responded Differently

**FIGURE 6:**
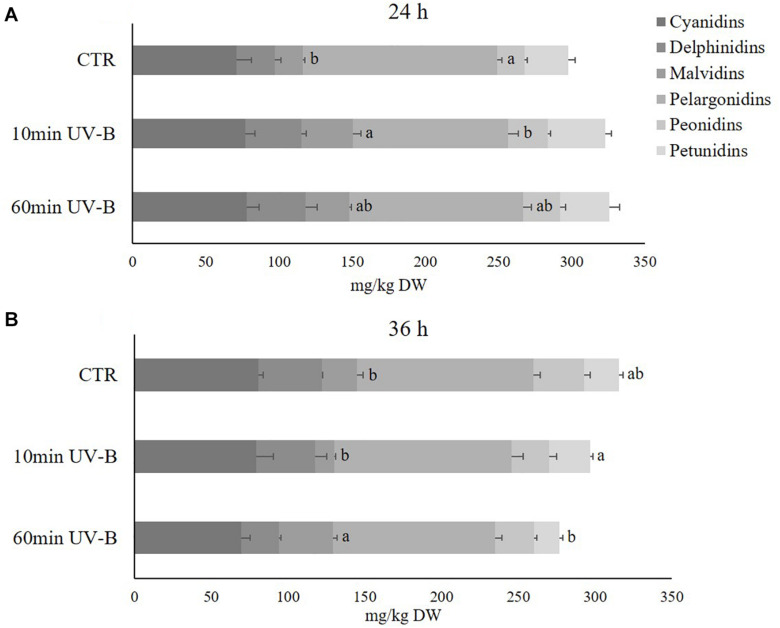
Concentration (mg/kg DW) of different anthocyanins (cyanidins, delphinidins, malvidins, pelargonidins, peonidins, and petunidins) in the pulp of peaches exposed to UV-B radiation for either 10 or 60 min and sampled after 24 h **(A)** and 36 h **(B)** from the exposure. Data are mean ± SE of five biological replicates. Different letters correspond to statistically significant differences according to one-way ANOVA followed by Tukey–Kramer *post hoc* test (*P* ≤ 0.05).

Anthocyanins belong to the second most abundant flavonoid class, and in the light of their great importance both for humans as antioxidant and health-promoting compounds, and for plants as colored, attractive pigments for pollinators and seed dispersers, our subsequent investigation was to elucidate whether and how the UV-B treatment might have influenced individual anthocyanins. We hypothesized that, although anthocyanins as whole class might not display significant variations after UV-B exposure, different compounds belonging to the same subclass might respond differently. Sixty-one different anthocyanins, including both glycosides and aglycones, were identified (Supplementary Table S3). Similar to analyses of flavonols, anthocyanins were classified in 17 cyanidins, 9 delphinidins, 7 malvidins, 11 pelargonidins, 8 peonidins, and 9 petunidins. The most representative anthocyanins in the pulp of peach fruit were the pelargonidins (133.2 and 115.2 mg/kg in the 24- and 36-h recovery time points, respectively) and the cyanidins (71.1 and 80.5 mg/kg in the 36-h recovery time point, respectively), followed by delphinidins (26 and 41 mg/kg in the 24- and 36-h recovery time points, respectively), petunidins (29.5 and 22.6 mg/kg in the 24- and 36-h recovery time points, respectively), peonidins (18.7 and 33.0 mg/kg in the 24- and 36-h recovery time points, respectively) and malvidins (18.9 and 22.6 mg/kg in the 24- and 36-h recovery time points, respectively) (Figure 6). Along the recovery time tested, they all underwent a slight increase in controls, especially for pelargonidins (+ 12.7 mg/kg), delphinidins (+ 11.1 mg/kg) and peonidins (+ 10.1 mg/kg), underlying the physiological modifications that such phenolic compounds might experience during storage. As observed for flavonols, the UV-B treatment was able to affect the concentration of the anthocyanin subclases differently. Indeed 24 h from the exposure, the UV-B-responsive anthocyanins were the malvidins and the pelargonidins. Malvidins underwent a significant increment in the 10-min UV-B treated samples (+ 87%). Pelargonidins, however, decreased significantly in the 10-min UV-B-irradiated peaches, while no modifications occurred after the 60-min treatment for both subclasses. At the 36-h recovery time points, the concentration of malvidins was still significantly affected by the irradiation, being 56% higher in the 60-min UV-B-treated fruit. Furthermore, the concentration of petunidins showed a trend of reduction in the 60-min UV-B-treated samples (-29%). Thus, although the total anthocyanin concentration did not show any variation (Figure 2), the anthocyanin profile was affected by the UV-B exposure.

#### UV-B Irradiation Reduced the Concentration of Anthocyanin Aglycones Without Affecting the Concentration of Glycosides

Since the ratio between glycosides and aglycones of flavonols was differentially modulated by UV-B (Table 1), the effect of UV-B on the ratio of anthocyanin glycosides and aglycones were determined (Table 2). First, also anthocyanin glycosides were more concentrated than their aglycones. Specifically, glycosides were on average 277 and 270% higher than aglycones in the 24 and 36 h recovery time points, respectively, compared to the control samples. Considering the UV-B treatments, both 10- and 60-min irradiations triggered a significant decrease in anthocyanin aglycones regardless of the recovery time point (-25 and -56% after 24 h, and -32 and -30% after 36 h, following 10- and 60-min UV-B-treatment respectively). On the other hand, anthocyanin glycosides were not significantly influenced by the UV-B radiation at any recovery time point.

**TABLE 2:** Concentration of total anthocyanin aglycones and glycosides in the UV-B-treated samples after 24 and 36 h from the UV-B exposure.

		Aglicones	Glycosides
		(mg/kg DW)	(mg/kg DW)
24 h	CTR	62.4 ± 2.4*Ab*	235.1 ± 16.8a
	10 min UV-B	46.5 ± 13.5*Bb*	287.6 ± 30.8a
	60 min UV-B	27.6 ± 11.5*Bb*	288.2 ± 34.8a
36 h	CTR	67.2 ± 2.3*Ab*	248.4 ± 4.5a
	10 min UV-B	45.6 ± 9.7*Bb*	224.3 ± 28.7a
	60 min UV-B	47.2 ± 23.4*Bb*	216.8 ± 18.2a

*Data are mean ± SE of three biological replicates. Different letters correspond to statistically significant differences according to one-way ANOVA followed by Tukey–Kramer *post hoc* test (*P* ≤ 0.05). Lowercase letters refer to the comparison between aglycones and glycosides, while uppercase letters refer to the comparison between the controls and the UV-B-treated groups in each recovery time point (24 or 36 h).*

### Molecular Analysis Revealed Activation of Genes Involved in UV-B-Signaling and Phenylpropanoid Biosynthetic Pathways

**FIGURE 7:**
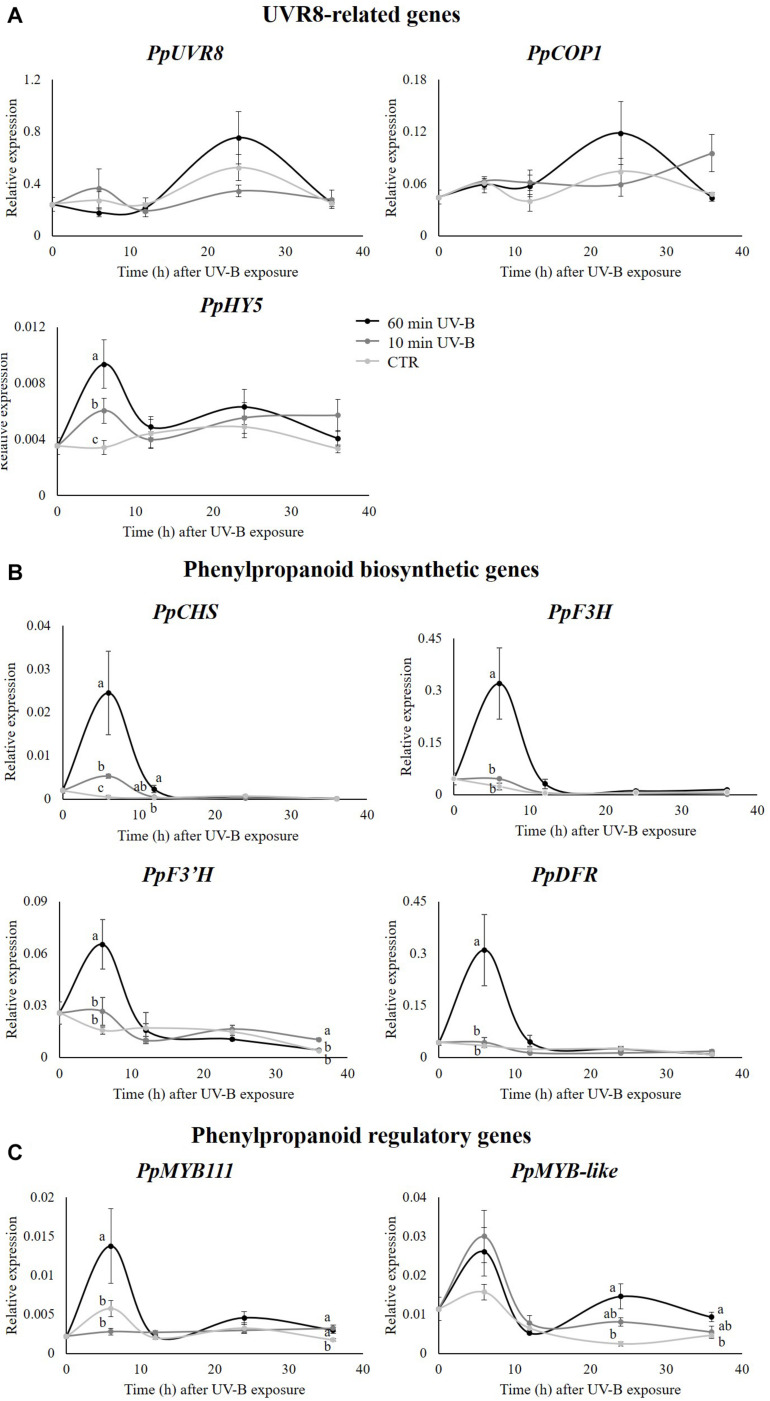
Transcript level of: **(A)** some genes involved in UVR8 pathway (UVR8, COP1, HY5), **(B)** some structural (CHS, F3H, F30H, DFR) and **(C)** some regulatory (MYB111, MYB-like) genes involved in phenylpropanoid biosynthesis in the pulp of peaches exposed to UV-B radiation for either 10 or 60 min, and sampled after 24 and 36 h from the exposure. Data are mean ± SE of five biological replicates. Different letters correspond to statistically significant differences according to one-way ANOVA followed by Tukey–Kramer *post hoc* test (*P* ≤ 0.05).

Once elucidated that UV-B radiation was effective in modulating the flavonoid profile, with emphasis for specific flavonoid subclasses, the next step was to investigate at the molecular level expression of genes involved in the phenylpropanoid pathway. Accordingly, the transcript abundance of three UVR8-signaling related genes (*PpUVR8*, *PpCOP1*, *PpHY5*), four phenylpropanoid biosynthetic (*PpCHS*, *PpF3H*, *PpF3*′*H*, *PpDFR*) and two regulatory (*PpMYB111*, *PpMYB-like*) genes were investigated. The results are reported in Figure 7. Concerning the UVR8-signaling related genes (Figure 7A), UV-B radiation did not induce any variation at the transcript level of *PpUVR8* and *PpCOP1*, with respect to any recovery time points, though both genes showed a similar (not significant) increase in their expression after 24 h from the 60-min UV-B exposure compared to the control level. On the other hand, transcription of *PpHY5* significantly increased after 6 h in a dose-dependent manner (+ 77 and + 174% following 10- and 60- min UV-B irradiation, compared to the control level, respectively). In the following recovery time points, *PpHY5* transcription in both control and UV-B-treated fruit was maintained in a steady state up to 36 h, without significant variations. Contrarily to the UVR8-signaling related genes, all the phenylpropanoid biosynthetic genes tested showed a consistent UV-B-induced upregulation, especially in the early time points after UV-B exposures (Figure 7B). Indeed, the expression of *PpCHS*, *PpF3H*, *PpF3*′*H* and *PpDFR* exhibited a noteworthy increase after 6 h from the 60-min UV-B treatment compared to the control level (+ 5024, + 1285, + 317, and + 803%, respectively). The 10-min treatment was also effective in overexpressing *PpCHS* after 6 h (+ 1009%) and the UV-B-induced upregulation of this gene was detected also after 12 h from the 60-min irradiation (+ 648%). Considering the late time points, the expression of the phenylpropanoid biosynthesis genes remained stable with no significant UV-B related variations, except for the *PpF3*′*H*, whose transcript level increased in the 10-min UV-B-exposed fruit after 36 h (+ 163%). Among the regulatory genes, the expression of *PpMYB111* showed a similar behavior to the phenylpropanoid biosynthetic genes. In fact, a consistent upregulation was observed in the 6-h recovery time point following the 60-min UV-B-exposure (+ 139%). After this early UV-B-induced upregulation, no significant differences were detected up to 24 h from the irradiation among the treated and untreated groups, while the UV-B-exposed samples showed a higher transcript abundance after 36 h from both the 10- and 60-min irradiation (+ 71 and + 86% compared to the control, respectively). *PpMYB-like* expression slightly differed from *PpMYB111*. Although a similar, UV-B-induced peak might be seen after 6 h in both the treated groups, such increase was not statistically significant. In contrast, the effect of UV-B radiation on *PpMYB-like* was detectable in the latter recovery time points, where a dose-dependent response was found. Its transcript level was 498 and 101% higher in the 60-min UV-B treated samples after 24 and 36 h from the irradiation, respectively, while in the 10-min treated samples, this gene showed only a trend of induction at the same recovery time points (Figure 7C).

## Discussion

### The UV-B-Driven Rearrangement of the Phenolic Profile in the Peach Pulp Differs From What Observed in the Skin

This work aimed to deeply investigate how the UV-B radiation can modify the phenolic profile in the pulp of peach fruit, focusing on the flavonoid subclasses, e.g., flavonols and anthocyanins. Besides, the UV-B-mediated responses of flavonols and anthocyanins glycosides and aglycones were studied, to determine whether such modulation might occur differently according to the linked sugar moieties. Lastly, a molecular analysis was conducted on several UVR8-signaling related and phenylpropanoid biosynthetic and regulatory genes, to understand if such modulation might be due to the UV-B photoreceptor, UVR8. Although several previous studies have deeply investigated the UV-B-induced modulation of phenolic profiles in many fruit (Luthria et al., 2006; Interdonato et al., 2011; Ruiz et al., 2016; Assumpção et al., 2018; Santin et al., 2018a, 2019) and vegetable species (Eichholz et al., 2012; Rajabbeigi et al., 2013), they principally analyzed just the outermost tissue, since it is directly exposed to the radiation and therefore it is most likely to be influenced by the UV-B exposure. Other studies used the whole organism, risking mixing different responses the UV-B irradiation might trigger on different tissues.

In the present work, the output of the CDA (Figure 1) revealed that the UV-B irradiations, both for 10 and 60 min, resulted in a strong modulation of the phenolic profile in peach pulp. In addition, since UV-B-treated and control groups were separated also in respect to recovery time points, it is reasonable to assume that also the storage time had an impact on the entire phenolic profile. A whole rearrangement of this latter was observed also in the peel of peaches exposed to similar UV-B treatments (Santin et al., 2019). Particularly, the phenolics on peach peel responded mostly after 36 h from the UV-B exposure, with 89% of the detected phenolics undergoing an increase (Santin et al., 2019). When the individual flavonoid classes were considered, a different effect was observed after 24- or 36-h recovery. After 24 h from the exposure, indeed, all the responsive flavonoid classes (flavones, dihydroflavonols, flavonols, and flavanols) exhibited a significant increase following the UV-B treatment, especially the 60-min irradiation. After 36 h, however, the significantly modulated classes were just the flavonols, which increased after the 60-min irradiation, and the flavanols, which decreased in all the UV-B-treated groups. This result was partially in contrast with what was observed by Santin et al. (2018a) in the peel of UV-B-exposed peaches, where, after a general decrease in most phenolic classes at 24 h from the UV-B exposure, a significant increase occurred 36 h from the irradiation, especially for dihydroflavonols, anthocyanins, and flavones. Besides, this effect was mainly visible in the 60-min UV-B-irradiated samples, which was the strongest treatment tested in both studies. Such discrepancy suggests a different responsiveness of the two tissues toward the UV-B radiation. As stated by Santin et al. (2019), in fact, the initial decrease in phenolic concentration in peach peel might be due to their consumption to counteract the potentially harmful UV-B-induced ROS. However, the later UVR8-induced activation of phenylpropanoid genes might have triggered the synthesis and accumulation of new phenolic compounds, as observed after 36 h. Recent studies (Santin et al., 2020) have shown that UV-B radiation does not penetrate peach peel. Thus, it is assumable that a direct UV-B-driven overproduction of ROS did not occur in the pulp. As a result, the phenolics in the pulp were not consumed by their ROS-scavenging activity but they rather increased due to the molecular activation of several genes involved in the phenylpropanoid biosynthesis and regulatory pathway, e.g., *PpCHS*, *PpF3H*, *PpF3*′*H*, *PpDFR* and some *PpMYB* genes, as reported in Figure 7.

### UV-B Radiation Affects the Expression of UVR8-Related and Phenylpropanoid-Related Genes in the Peach Pulp

Influence of UV-B radiation on the level of genes involved in the flavonoid biosynthesis was elucidated in many fruit species, such as peach (Scattino et al., 2014; Zhao et al., 2017; Santin et al., 2019), apple (Ubi et al., 2006; Bai et al., 2014), and tomato (Giuntini et al., 2008; Calvenzani et al., 2010; Catola et al., 2017). In contrast to the present study, however, the transcript abundance in the cited works was investigated in the fruit tissue directly exposed to the UV-B radiation, thus likely to be influenced by the exposure. Considering that UV-B radiation was found to be blocked by the peach peel (Santin et al., 2020), the UV-B-mediated activation of phenylpropanoid-related genes described in this manuscript was probably not directly influenced by the UV-B radiation. Indeed, the *PpUVR8* and *PpCOP1* genes did not show any significant variation in response to the treatments, while the activation of *PpCOP1* was observed in peach peel (Santin et al., 2019). It is reasonable to assume that the increased transcription level of the flavonoid genes in the pulp was due to a chemical interplay between the outermost, UV-B-exposed peel and the pulp below. A possible candidate playing this role might be the *PpHY5*, which was in fact overexpressed in both the peel (Santin et al., 2019) and the pulp of the UV-B-treated samples, particularly after 6 h from the irradiation. HY5, member of the basic leucine zipper (bZIP) transcription factors, represents a key factor in the UVR8 signaling pathway, whose expression was found to be upregulated by the UVR8-COP1 complex after the UV-B perception (Ulm et al., 2004; Brown et al., 2005; Huang et al., 2012; Binkert et al., 2014). Once HY5 is stabilized in the cell, it promotes its own expression under both visible and UV-B radiation through the binding to its own promoting sequence (Abbas et al., 2014; Binkert et al., 2014). HY5 was also found to be able to translocate from the plant shoots to the roots, in order to promote and optimize the root nitrate absorption according to the environmental light conditions (Lillo, 2008; Nunes-Nesi et al., 2010; Chen et al., 2016). Though no studies have investigated a possible transport of the HY5 transcription factor among fruit tissues, it might be possible that the overproduced HY5 in the peach peel after the UV-B exposure might have reached the pulp below, triggering its own transcription and, in turn, the transcription of some flavonoid-related genes (Brown et al., 2005; Brown and Jenkins, 2007). This might have led to the accumulation of flavonoids detected after 24 h from the irradiation, such as flavanols, flavonols, and flavones. A further hypothesis that might explain the activation of UVR8- and flavonoid-related genes in the peach pulp although UV-B-protected by the peel could be that the UVR8 in the pulp is activated by wavelengths longer than UV-B, which can weakly penetrate through the peel. Accordingly, recent evidences (Rai et al., 2020) have shown that UVR8 is involved in the perception of both UV-B and UV-A up to 350 nm. However, although UV-B narrowband lamps used in this study has a weak emission within the UV-A range, the transmittance across the peel is below 1% considering both yellow and red peel portions (Santin et al., 2020), thus it is unlikely that such irradiance is able to trigger the UVR8 pathway. In addition, while other studies identified a light gradient within tissues (Seyfried and Fukshansky, 1983; Day et al., 1993), recent work in the model plant Arabidopsis demonstrates that multiple cell types contribute to UV-B signaling and that UV-B can reach the endodermis and pith of Arabidopsis stems, hypocotyls and cotyledons where the UVR8 signaling leads to local flavonoid accumulation (Bernula et al., 2017; Vanhaelewyn et al., 2019a, b).

### Glycosylated Flavonols Were Differently Influences by UV-B Exposure

Responsiveness of flavonols, kaempferol glycosides, toward supplemental UV-B radiation was found also in the leaves of *Brassica napus* L. cultivars, Paroll (Olsson et al., 1998). Concentration of kaempferol glycosides in plants grown under 800 μmol m^–2^ s^–1^ with additional 13 kJ m^–2^ day^–1^ UV-B radiation was 35% higher in the UV-B-treated plants. Moreover, the concentration of kaempferol-3-sophoroside-7-glucoside increased by 76% compared to the unirradiated plants. Interestingly, in our study, the same kaempferol glycoside was found to be accumulated in the 60-min UV-B-treated fruit after 36 h from the end of the exposure. However, other kaempferol glycosides found by Olsson et al. (1998), such as the kaempferol 3-2”’-sinapoylsophoroside-7-glucoside, were not similarly increased, underlying the differential behavior that different kaempferol glycosides might have toward UV-B exposure also in other plant species. A marked variation in the response to UV-B irradiation among the different flavonols was found also in chive (*Allium schoenoprasum* L.) cultivated under 15 mol m^–2^ d^–1^ PAR supplemented with 2 kJ m^–2^ d^–1^ UV-B radiation (Grubmüller et al., 2004). Particularly, the highest increase was observed for quercetins followed by isorhamnetins and kaempferols. Increase in flavonols was also reported in *Centella asiatica* L. Urban. grown under low (455 μmol m^–2^ s^–1^) and high low (835 μmol m^–2^ s^–1^) PAR, supplemented with 0.3 W m^–2^ UV-B radiation (Müller et al., 2013). Indeed, Müller et al. (2013) found that both low and high PAR conditions determined a significant increase in flavonol concentration only when supplemented with UV-B radiation. Another study on peeled onion bulbs exposed to a combination of UV-A, -B and -C radiations (18 W m^–2^) showed a 2-fold increase in flavonol concentration (Rodov et al., 2010). Also in the peel of peach fruit, 3- and 6-h UV-B treatments (1.36 W m^–2^) were effective in inducing a significant, strong accumulation of flavonols after 24 h from the exposure by 174 and 76%, respectively (Santin et al., 2018b). Particularly, both the kaempferols detected in that work, the 3-rutinoside and the 3-galactoside, underwent a significant increase in the UV-B-treated samples. Similarly, the kaempferol 3-rutinoside was found to be positively influenced by the UV-B radiation also in the present study. Besides, Santin et al. (2018b) observed an increase also in isorhamnetin concentration in the 3-h UV-B-treated fruit considering all the individual isorhamnetins detected, which were the 3-rutinoside, the 3-galactoside and the 3-glucoside. In the current work, isorhamnetin 3-rutinoside was likewise significantly increased after 24 h from the 60-min UV-B exposure.

### The Different Anthocyanins Responded Differently to the UV-B Treatment, Although Total Anthocyanin Concentration Was Unchanged

Despite no significant variations were observed in the concentration of total anthocyanins, regardless of the recovery time point, this result derived from a differential influence played by UV-B radiation on anthocyanin subclasses. Indeed, a deeper investigation revealed that malvidins and pelargonidins significantly increased and decreased 24 h after the 10-min UV-B-treatment, respectively. After 36 h, the influenced anthocyanins were malvidins and petunidins, which decreased and increased in the 60-min UV-B-exposed peaches, respectively. These results were in contrast to what was observed in the peel of UV-B-treated peach fruit. Santin et al. (2018a) found a consistent accumulation of anthocyanins 36 h after both the 10- and the 60-min UV-B exposure (1.92- and 2.53-fold increase, respectively), and also Santin et al. (2018b) displayed a higher cyanidin-3-glucoside concentration following a 3- and 12-h UV-B-treatment. Anthocyanins play a key role in photoprotection against high-energy radiations, such as UV (Solovchenko and Schmitz-Eiberger, 2003; Guo et al., 2008; Schreiner et al., 2012). A stimulation in the anthocyanin biosynthesis following UV-B exposure, which was widely elucidated in many previous works (Marais et al., 2001; Kataoka and Beppu, 2004; Higashio et al., 2005), might not have occurred in the pulp because, being completely UV-B-shielded, did not need to boost up its defenses by accumulating newly-synthesized anthocyanins. Furthermore, in UV-B-treated apple fruit, biosynthesis of flavonols occurred faster than anthocyanins biosynthesis, leading to a higher accumulation of flavonols compared to what was observed for anthocyanins (Ban et al., 2007; Hagen et al., 2007).

### UV-B Exposure Increased the Glycosides/Aglycones Ratio of Flavonols and Anthocyanins

In the present research, the UV-B treatments revealed a significant increase in the glycosides/aglycones ratio of flavonols and anthocyanins. While the glycosilated ones increased the aglycone concentration decreased. Besides, such transient effect on flavonols was noticeable only after 24 h in both UV-B treatments. Most flavonoids are normally glycosilated (Tohge et al., 2017) providing several advantages, such as increased solubility and, consequently, easier storage and reduced transportation across cell membranes (Vetter, 2000; Kytidou et al., 2020). Thus, it might be that the increased glycosides/aglycones ratio was due to the necessity of the cells to optimize the storage of the overproduced flavonoids by glycosylation. Furthermore, the stability and the bioactivity of glycosylated phytochemicals are related to the position where sugar moieties are linked, as well as the type of linkage (*O*- versus *C*-) and of sugars (Kytidou et al., 2020). Unfortunately, literature on UV-B-induced modification of glycosylation pattern is scarce. Neugart et al. (2014) found that kale plants exposed to five daily doses of 0.25 kJ m^–2^ d^–1^ UV-B radiation differentially accumulated quercetins and kaempferols depending on the sugar moiety and the degree of acetylation (whether non-, mono-, or di-acetylated flavonol). Similarly, in this work, no correlation was observed between either the type and number of sugar moieties linked to the flavonoid molecule (mono-, di-, or tri-saccharides) and the most UV-B-responsive kaempferols or isorhamnetins.

## Conclusion

The present study provides evidence that, although UV-B radiation does not penetrate the peel of peach fruits, it rearranges the flavonoid classes in the pulp underneath. Besides, such UV-B-induced changes of the flavonoid profiles depend on both the flavonoid class and the recovery time, and are particularly pronounced when considering the glycosides and the aglycones separately. Since UV-B-driven metabolic changes on fruits and vegetables are strictly UV-B dose- and cultivar-dependent, further “-omics” studies on other plant species are highly encouraged to extend the knowledge on how to exploit the UV-B radiation to enhance the nutraceutical value on plant-based food.

## Data Availability Statement

All datasets generated for this study are included in the article/Supplementary Material.

## Author Contributions

AR and M-TH designed the research. MS, AC, LL, GR, and BM-M carried out the experiments, analyzed the data, and wrote the manuscript. AR, LL, and M-TH helped to draft the manuscript and revised the manuscript. All authors read and approved the final manuscript.

## Conflict of Interest

The authors declare that the research was conducted in the absence of any commercial or financial relationships that could be construed as a potential conflict of interest.

## Footnotes

1 https://www.rosaceae.org/species/prunus_persica/genome_v2.0.a1

2 http://phenol-explorer.eu/
