# Supplementary material for: Beyond the Visible and Below the Peel: How UV-B Radiation Influences the Phenolic Profile in the Pulp of Peach Fruit. A Biochemical and Molecular Study
Source: Front Plant Sci. 2020 Oct 30;11:579063. doi: 10.3389/fpls.2020.579063 (PMC7661749; doi:10.3389/fpls.2020.579063)
Supplement: Supplementary file 2 [file Table_2.DOCX]

**Tab. S2** List of primers used in this work.

| Gene | Forward (5'-3') | Reverse (3'-5') | AGI number | gDNA fragment length (bp) | cDNA fragment  length (bp) | PCR efficiency  E = 10^−1/S^-1 |
| --- | --- | --- | --- | --- | --- | --- |
| *PpEIF4A* | TCG TGA GGA TCA GCG CAT TC | TGG CAT TGT AGC AGA GAA CAC | Prupe.1G010400 | 340 | 229 | 0.91 |
| *PpTUB9* | GTT GAG CCA TAC AAT GCT ACC | GCT TCC TAA GGT CAG AGT TCA | Prupe.4G095300 | 402 | 223 | 0.99 |
| *PpUBQ5* | CTC AGG AAG GAG TGC CCC A | TAA GAA CAC GGT TCC AAA TCC T | Prupe.3G004600 | 229 | 207 | 0.91 |
| *PpCHS* | AGG GTG CTC GTG TTC TCG TT | CGG GAA GGA TGG TTT GGG CT | Prupe.1G002900 | 201 | 201 | 0.97 |
| *PpF3H* | GAA GGA GCC TTC GTG GTC AA | TTG GAA TGT GGC TAT GGA CAG | Prupe.7G168300 | 595 | 124 | 0.91 |
| *PpF3’H* | GAG GAT GCT GAC GGT GAG G | GGT GGC GAA GGA GTT CTG C | Prupe.5G203600 | 879 | 136 | 0.94 |
| *PpDFR* | GAC GAA ACC GAC TGG AGC GA | TGG CAT GGA TGG CAT GAG AAA T | Prupe.1G376400 | 296 | 183 | 1.20 |
| *PpMYB111* | CTT ATT GCT GGA AGG TTG CCA | AGG CTA AAC CAG ATC ATT CCT C | Prupe.3G158700 | 130 | 130 | 1.31 |
| *PpMYB-like* | CAC CAG AAC TTT GGA TCA CAG | GGT GAC TGA TCC ATC TCC TG | Prupe.7G016700 | 176 | 340 | 0.91 |
| *PpCOP1* | GAA GAA CTT CGT AGG TCT TAC A- | TGG TTC CCT GAC TGT TAG CAG | Prupe.5G031300 | 308 | 232 | 0.82 |
| *PpHY5* | AGC AAG CAA GGG AGA GGA AGA | CCT CTC CGG CTT GCC GTT G | Prupe.1G478400 | 1114 | 163 | 0.91 |
| *PpUVR8* | GGT GTC ACC ATC AGA GAG ATA | TCT TAA ATA CTA GGG CAT CCC A | Prupe.4G277200 | 848 | 159 | 0.94 |

^S^ refers to the slope of the standard curve.
